# A comparison of methods for calculating general practice level socioeconomic deprivation

**DOI:** 10.1186/1476-072X-5-29

**Published:** 2006-07-04

**Authors:** Mark Strong, Ravi Maheswaran, Tim Pearson

**Affiliations:** 1 Rotherham Primary Care Trust, Oak House, Moorhead Way, Bramley, Rotherham, S66 1YY, UK; 2 Public Health GIS Unit, School of Health and Related Research, The University of Sheffield, Regent Court, 30 Regent Street, Sheffield S1 4DA, UK

## Abstract

**Background:**

A measure of the socioeconomic deprivation experienced by the registered patient population of a general practice is of interest because it can be used to explore the association between deprivation and a wide range of other variables measured at practice level. If patient level geographical data are available a population weighted mean area-based deprivation score can be calculated for each practice. In the absence of these data, an area-based deprivation score linked to the practice postcode can be used as an estimate of the socioeconomic deprivation of the practice population. This study explores the correlation between Index of Multiple Deprivation 2004 (IMD) scores linked to general practice postcodes (main surgery address alone and main surgery plus any branch surgeries), practice population weighted mean IMD scores, and practice level mortality (aged 1 to 75 years, all causes) for 38 practices in Rotherham UK.

**Results:**

Population weighted deprivation scores correlated with practice postcode based scores (main surgery only, Pearson r = 0.74, 95% CI 0.54 to 0.85; main plus branch surgeries, r = 0.79, 95% CI 0.63 to 0.89). All cause mortality aged 1 to 75 correlated with deprivation (main surgery postcode based measure, r = 0.50, 95% CI 0.22 to 0.71; main plus branch surgery based score, r = 0.55, 95% CI 0.28 to 0.74); population weighted measure, r = 0.66, 95% CI 0.43 to 0.81).

**Conclusion:**

Practice postcode linked IMD scores provide a valid proxy for a population weighted measure in the absence of patient level data. However, by using them, the strength of association between mortality and deprivation may be underestimated.

## Background

Socioeconomic deprivation is strongly linked to both health and health care access [1], and is therefore of interest to policy makers, commissioners, public health professionals and researchers.

A measure of the socioeconomic deprivation experienced by the registered patient population of a general practice is of interest because it can be used to explore the association between deprivation and a wide range of other practice characteristics. In particular, a practice level measure of deprivation can help inform the answer to the question: "is the care delivered across this district equitable?"

Previous research has examined the association between practice deprivation and referral rates [2], prescribing rates for coronary heart disease [3,4], access to cardiac services [5], out of hours activity [6], achievement against the UK general practitioner Quality and Outcomes Framework contract [7-9], voluntary engagement in the UK general practitioner Personal Medical Services contract [10], and the prevalence and outcome of depression [11]. Some measure of deprivation is also necessary in order to adjust for the confounding effect of socioeconomic variation when exploring other practice level associations [12]. In each of these studies, the measure of socioeconomic deprivation chosen was based on an area level deprivation index, rather than on individuals' socioeconomic status.

Area level deprivation measures are commonly used because they do not require individual level socioeconomic status data, other than those obtained from routine sources such as the National UK Census. Commonly used area deprivation measures include the Carstairs [13], Townsend [14], and Jarman Underprivileged Area indexes [15], and more recently the Index of Multiple Deprivation [16].

Individuals can be linked via their residential postcode to a geographical area (for example an enumeration district or census super output area) and therefore assigned a deprivation score representative of the area in which they live. The mean of the deprivation scores linked to the residencies of all patients registered with a general practice can then be taken as a summary measure of the socioeconomic deprivation experienced by that general practice population. However, the difficulty with this method is the need for patient level geographical data: the location of each of the registered patients needs to be known in order to assign a deprivation score. Although these data are used routinely within Primary Care Organisations (PCOs) for needs assessment, planning and evaluation, they are not easily accessible to researchers working in another health services organisation (for example a different PCO or the national Health Protection Agency), or outside of the health service [17].

It is straightforward, however, to assign a deprivation score to a practice based on the practice postcode alone, and some investigators have used this approach [9,11,12]. The assumption underlying this method is that the level of deprivation experienced by the population in the locality of the practice is a reasonable proxy for the level of deprivation of the whole registered practice population. Given, however, that the practice population will live not only in the immediate locality of the practice, but in the surrounding areas as well, how valid is this assumption?

In this study we examined the correlation between practice postcode linked deprivation scores, and practice population weighted deprivation scores. We also examined the correlation between practice level mortality and deprivation, using scores calculated by the different methods.

## Methods

In 2004 the Office of the Deputy Prime Minister in the UK commissioned the construction of a deprivation index to cover the whole of England at Census Lower Layer Super Output Area (LSOA) level [18], based on 2001 census area geography. The resulting Index of Deprivation (ID 2004) has seven domains that reflect the multi-faceted nature of socioeconomic disadvantage: income, employment, health and disability, education, housing, environment and crime. These domains along with a combined measure, the Index of Multiple Deprivation (IMD 2004), are published at LSOA level in a downloadable table [19].

We calculated three measures of practice level deprivation. In the first method we used the All Fields Postcode Directory to link the postcode of the main surgery address to its LSOA [20], and hence to an IMD score. The second method took into account any branch surgeries that the practice operated, linking the postcodes of the main and any branch surgeries to their respective LSOAs, and then calculating the average of the LSOA linked IMD scores. Both these methods took no account of the residencies of the registered patient population. The third method used LSOA level counts of patients registered with each practice to calculate a mean IMD score, weighted for the proportion of the registered population living in each LSOA. This population weighted method took no account of the location of the surgery building or buildings.

We explored the correlation between the deprivation scores calculated by the three different methods using the Pearson correlation coefficient. We also explored the differences in correlations between the three measures of practice level deprivation and all cause mortality in those aged 1 to 75, directly standardised for age and sex using the European Standard Population (pooled 2003 and 2004 deaths; data from the Public Health Mortality File, Office for National Statistics). We used Fisher's Z transformation method to calculate correlation coefficient 95% confidence intervals [21].

## Results

Rotherham is a mixed urban and rural district in the South Yorkshire coalfields. It is an area of relatively high socioeconomic deprivation, with a mean IMD score of 28.2 (ranked 63^rd ^most deprived out of England's 354 local authority areas) [19]. Rotherham's mortality rate is greater than that for both the region and the country as a whole. (Pooled 2002–4 deaths from all causes, all ages, directly standardised per 100,000 European Standard Population, Rotherham: 886.5, 95% confidence interval 859.0 to 914.1; Yorkshire and Humber Region: 827.5, 95% confidence interval 821.6 to 833.4; England: 784.9, 95% confidence interval 783.1 to 786.7) [22].

In January 2004 Rotherham had a population of approximately 251,000 patients registered to 39 general practices. One of the practices was a small specialist practice, managed by the primary care trust, which cared specifically for asylum seekers and homeless people. Its registered population was less than 1% of the total Rotherham registered population and it was not included in this analysis. Nine of the 38 remaining practices had a single branch surgery and four of the practices had two branch surgeries. All surgery postcodes were matched to the LSOA containing the postcode centroid, and hence to a corresponding IMD score. The population weighted IMD scores took into account 99% of Rotherham registered patients. The remaining 1% had missing postcode data and were not able to be matched to a LSOA. The pooled total number of deaths (aged 1 to 75 years) in Rotherham during 2003 and 2004 was 1756.

The Pearson correlation coefficient for the correlation between main practice postcode based IMD scores and population weighted IMD scores was 0.74 (95% confidence interval 0.54 to 0.85). This correlation marginally increased to 0.79 (0.63 to 0.89) when the locations of branch surgeries were added in, but the confidence intervals were overlapping.

Directly standardised all cause mortality in those aged 1 to 75 correlated with deprivation. The correlation with the population weighted measure was 0.66 (95% confidence interval 0.43 to 0.81); with the main practice postcode based measure it was 0.50 (0.22 to 0.71); and with the main and branch surgery postcode based measure it was 0.55 (0.28 to 0.74). All confidence intervals were overlapping.

## Discussion

### Main finding of this study

We found significant correlations between a practice postcode based deprivation measure (with or without taking into account branch surgeries), and a practice population weighted deprivation measure.

We also found a significant correlation between all cause mortality and deprivation, as we would expect [23]. Although correlation coefficients were greater with the registered population weighted rather than the practice postcode based IMD scores, confidence intervals were overlapping, indicating that the difference may not be statistically significant.

### What is already known on this topic

Previous studies have compared the effects of using deprivation scores derived for geographical areas of varying sizes [24,25], but we found no study that directly compared a measure calculated from a single postcode with a population weighted measure.

### What this study adds

**Figure 1:**
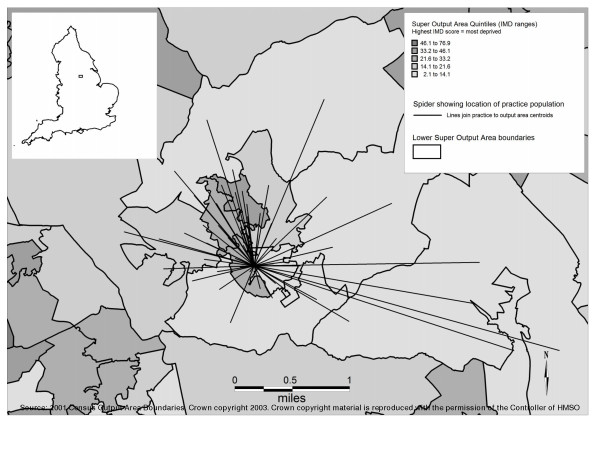
**Spider map showing locations (census output area centroids) of registered population for one GP practice**. Only output areas with greater than 10 registered patients are included. Inset map shows location of Rotherham within England.

Figure 1 illustrates why an IMD score based on the practice postcode alone will only ever be an approximation of the area-based deprivation of the practice population as a whole. Patients live within a number of different LSOAs, each one experiencing a different level of deprivation. This geographical distribution of patients across several LSOAs is typical of all practices in Rotherham.

If it is assumed that practices are not located in areas systematically more or less deprived than the surrounding area, then the error introduced by using the practice postcode measure of deprivation instead of the population weighted measure should be random. This random error is likely to reduce the strength of association between the deprivation measure and a health outcome such as mortality.

In order to use the practice postcode alone as the basis of a practice population weighted area-based deprivation measure a more sophisticated model could be envisaged: one in which both the level of deprivation of all surrounding small areas, and the geographical distribution of patients around a "typical" general practice are taken into account. Even then, this model will still only be an approximation, but the resulting measure may be more useful than the simple postcode assigned measure in those situations when patient registration data are unavailable.

### Limitations of this study

Our study looked only at 38 practices within one PCT. Although Rotherham is a mixed urban and rural area, the distribution of both small area level deprivation and of patients around practices will not be the same as that in all other areas in the UK. The relatively small number of practices also meant that confidence intervals for the correlation coefficients were wide.

A deprivation measure that is assigned to a group of people (for example the population of a general practice, or a LSOA) is only an aggregate measure of the experience of that group. Any single individual within the group may experience a quite different level of deprivation, and an association seen at the group level, say with mortality, may therefore not apply at the individual level. Assuming it does apply is known as the ecological fallacy.

The problem of the ecological fallacy is always an important consideration when using data at a small area or group level, and is well described in the literature (for example see [26]). Having said this, area level associations are interesting and important whether they reflect individual level associations or not, and indeed an ecological rather than individual level analysis is in many circumstances more appropriate [27]. Deprivation has been shown to operate independently as a risk factor for poor health at both the area level and the individual level [28], thereby necessitating the use of ecological analyses if we are to fully understand the interplay between socioeconomic factors and health.

## Conclusion

Practice postcode linked IMD scores are quick and easy to calculate, and provide a valid proxy for a population weighted measure in the absence of patient level data. However, the strength of association between ill health and deprivation may be underestimated if this method, rather than a population weighted approach, is used.

## Competing interests

The author(s) declare they have no competing interests.

## Authors' contributions

MS conceived the study, analysed the data and wrote the first draft of the manuscript. RM refined the study question and critically revised the manuscript. TP provided advice regarding the analysis and critically revised the manuscript. All authors have read and approved the final manuscript.
